# Association of lifestyle factors with mortality risk in people with chronic obstructive pulmonary disease: a prospective cohort study

**DOI:** 10.3389/fmed.2025.1527577

**Published:** 2025-04-30

**Authors:** Yue Zhang, Yue Ren, Zhenhua Li, Yaohua Yu, Xia Xu, Jianping Ye, Hua Zhang

**Affiliations:** ^1^Department of Pulmonary and Critical Care Medicine, Zhengzhou Central Hospital Affiliated to Zhengzhou University, Zhengzhou, China; ^2^Institute of Trauma and Metabolism, Zhengzhou Central Hospital Affiliated to Zhengzhou University, Zhengzhou, China; ^3^China Institute for Radiation Protection, Taiyuan, China; ^4^China Institute of Atomic Energy, Taiyuan, China; ^5^Tianjian Laboratory of Advanced Biomedical Sciences, Academy of Medical Sciences, Zhengzhou University, Zhengzhou, China

**Keywords:** Life’s Essential 8, mortality risk, chronic obstructive pulmonary disease, lifestyle, NHANES

## Abstract

**Background:**

Which lifestyle will benefit patients with chronic obstructive pulmonary disease (COPD) has becoming a hot topic in recent years. However, there are currently no recommendations. Life’s Essential 8 (LE8) includes 8 metrics (BMI, non-HDL cholesterol, blood pressure, blood glucose, physical activity, diet, sleep duration, and nicotine exposure), which are considered the foundation of maintaining a healthy life. Here, we aimed to explore the relationship between LE8 and mortality risk in patients with chronic obstructive pulmonary disease (COPD), which may provide advice on how to live better for these patients.

**Methods:**

Participants were from the National Health and Nutrition Examination Survey 2003–2018 at baseline linked to the 2019 National Death Index records. Cox proportional hazards regression models were used to explore the relationship between the LE8 score and mortality risk. All analyses were adjusted for survey design and weighting variables.

**Results:**

We included 1,593 participants with COPD, representing 9,208,187 US patients. During a median follow-up of 5.8 years, compared with patients with low LE8 scores, those with moderate and high score presented decreased all-cause mortality (both log-rank *p* < 0.05) and increased 10-year survival rates (63.6, 72.4 and 89.9%, respectively). Patients in the high (HR 0.19, 95% CI 0.09–0.40) and moderate (HR 0.61, 95% CI 0.43–0.86) score groups had a lower risk of all-cause mortality after adjusted for confounders. Similar results were observed in high (HR 0.53, 95% CI 0.32–0.88) and moderate (HR 0.68, 95% CI 0.50–0.91) HB score groups. Among the 8 metrics, physical activity, sleep health, nicotine exposure and blood glucose were important contributors to decreased mortality risk. And there were approximately linear dose–response relationships between LE8 and HB score with all-cause mortality risk.

**Conclusion:**

The LE8 score was inversely associated with all-cause mortality risk of patients with COPD. Changing lifestyles to improve LE8 scores may be an effective strategy to benefit these patients.

## 1 Introduction

Chronic obstructive pulmonary disease (COPD) is a heterogeneous chronic airway disease characterized by persistent airflow restrictions due to abnormalities of the airways or alveoli. It is one of the most common chronic respiratory disorders (1) and is a leading cause of death and disability worldwide (2). Although therapeutic strategies for COPD keep have improved recent years, there is still no good answer to how patients with COPD should manage their lifestyles and whether they can benefit from a healthy lifestyle.

Life’s Essential 8 (LE8) is a scoring system recently introduced by American Heart Association (AHA) to reflect cardiovascular health (CVH) (3), which can be compromised by COPD and, in turn, impact the outcomes of patients with COPD (4, 5). Although it was developed to evaluate CVH, many metrics, among them, have been proven to be closely associated with COPD development. For example, high consumption of processed red meat increases the risk of COPD by 40% compared with lower consumption (6). Higher blood glucose contributes to accelerated FEV1 decline (7). Nevertheless, previous studies mainly investigate the relationship between individual lifestyle factors and COPD. In contrast, LE8 not only is more comprehensive but also provides specific recommendations. However, it is uncertain whether these proposals can benefit patients with COPD.

In the present study, we aimed to explore the association of LE8 score with COPD mortality risk by using the National Health and Nutrition Examination Survey (NHANES) data. These findings may provide a combined and healthy lifestyle pattern to help patients with COPD live better.

## 2 Methods

### 2.1 Study population and demographic characteristics

NHANES is an ongoing, national and cross-sectional survey designed to assess the health and nutritional status of adults and children in the United States. This study was approved by the National Center for Health Statistics Research Ethics Review Board, and all participants provided written informed consent.

The population for this research consisted of 8 consecutive cycles of NHANES from 2003 to 2018. COPD were defined based on questionnaire items that is “Has a doctor ever told you that you have chronic bronchitis?” or “Has a doctor ever told you that you have emphysema?” and lung function tests which were performed only in three NHANES cycles (2007–2008, 2009–2010 and 2011–2012). Among the 25,934 participants, 2,189 aged 40–80 years old who answered yes to one of the above questions or whose FEV1/FVC was <70% after bronchodilator use was included. A total of 596 participants with missing data for LE8 elements or outcomes were excluded. Demographic information was collected via questionnaires.

### 2.2 Measurement of LE8

LE8 scoring algorithm consists of 4 health factors (HF) (BMI, non-high-density-lipoprotein cholesterol, blood glucose, and blood pressure) and 4 health behaviors (HB) (diet, physical activity, nicotine exposure, and sleep duration). Each metric was scored from 0 to 100 points according to reported algorithms (3) which can also be found in Supplementary Table. The total points were the average of the 8 metrics. Participants were grouped into high score (80–100), moderate score (50–80) and low score (0–50) groups according to the AHA recommendation.

### 2.3 Definition of mortality

Mortality follow-up data until December 31, 2019 were obtained from the National Death Index. The data were linked to baseline data from NHANES 2003–2018 on the basis of ID number.

### 2.4 Statistical analysis

All analyses were adjusted for survey design and weighting variables according to the analytical guidelines of the NHANES^1^. Baseline characteristics were characterized in terms of LE8 score. Continuous variables were shown as the means, and standard errors (SEs) and categorical variables were presented as percentages (weighted numbers).

The number of follow-up person-years was estimated from baseline to the end of the study, death or loss to follow-up, whichever came first. Kaplan–Meier survival curves were generated for to calculate the cumulative mortality of the three groups, and differences were examined via the log-rank test. Survey-weighted Cox proportional hazards regression models were used to explore the relationships between total LE8 score, HB score, HF score or single metric score (low level as the reference) and all-cause mortality. In the univariable model, no covariates were adjusted. Model 1 was adjusted for age, and model 2 was adjusted for age and education. The associations and interactions between LE8 and mortality in patients with COPD in different populations were investigated. All of the above analyses were performed via SAS version 9.4 (SAS Institute, Cary, NC) via the “SURVEY” procedures.

The dose–response association of total LE8 score and HB score with all-cause mortality were evaluated by restricted cubic spline models. The analysis was performed with R 4.3.0 (Core Team, Vienna, Austria) mainly via the rms package, the svydesign function and the svycoxph function.

## 3 Results

### 3.1 Baseline characteristics

The study included 1,593 participants aged 40–80 years, representing 9,208,187 individuals in the US with COPD. The characteristics with weighted population numbers were shown in Table 1. Among the participants, 5.6% (representing 517,828 patients) and 21.0% (representing 1,930,507 patients) had high and low LE8 scores, respectively. The mean age of the study participants was 61.9 years, and 51.4% were male. At baseline, participants with a high LE8 score were more likely to be women, and more likely to have a higher level of educational attainment and family income.

**Table 1:** Baseline characteristics of the study population.

Characterisics	Low LE8 score *N* = 359	Moderate LE8 score *N* = 1,034	High LE8 score *N* = 82	Total *N* = 1,593
Weighted *N*	1,930,507	6,759,852	517,828	9,208,187
Age strata, mean (SE)	59.71 (0.62)	62.42 (0.47)	63.48 (1.57)	61.91 (0.38)
Sex, % (Weight *N*, in thousand)				
Male	52.29 (1009)	52.59 (3555)	47.31 (170)	51.41 (4734)
Female	47.71 (921)	47.41 (3205)	52.69 (348)	48.59 (4474)
Race/ethnicity, % (Weight *N*, in thousand)				
NH White	76.63 (1479)	82.12 (5551)	88.62 (459)	81.34 (7490)
NH Black	10.73 (207)	6.62 (447)	4.63 (24)	7.37 (678)
Other Hispanic	2.73 (53)	2.91 (197)	1.78 (9)	2.81 (259)
Mexican-American	2.01 (39)	2.23 (151)	0.41 (2)	2.08 (192)
Other	7.91 (153)	6.12 (414)	4.55 (24)	6.40 (590)
Poverty ratio, % (Weight *N*, in thousand)				
< 1.3	52.64 (950)	24.87 (1575)	9.14 (40)	29.92 (2565)
1.3–3.5	33.67 (608)	38.68 (2450)	30.68 (133)	37.23 (3191)
>3.5	13.69 (247)	36.45 (2309)	60.18 (260)	32.85 (2816)
Education levels, % (Weight *N*, in thousand)				
High school or less	63.50 (1226)	51.33 (3470)	19.92 (103)	52.11 (4799)
Some college or associates degree	27.56 (532)	29.94 (2024)	19.07 (99)	28.83 (2655)
College graduate or above	8.94 (173)	18.72 (1265)	61.00 (316)	19.04 (1754)
Marital status, % (Weight *N*, in thousand)				
Coupled	57.32 (1107)	65.27 (4412)	76.43 (396)	64.23 (5914)
Single or separated	42.68 (824)	34.63 (2341)	23.57 (122)	35.69 (3287)
LE8 score, mean (SE)	40.81 (0.54)	64.27 (0.36)	84.88 (0.59)	60.51 (0.52)
HB score, mean (SE)	37.16 (1.07)	62.20 (0.68)	85.28 (1.00)	58.24 (0.70)
HF score, mean (SE)	44.46 (1.36)	66.35 (0.67)	84.48 (1.43)	62.78 (0.71)

SE, standard error; LE8, Life’s Essential 8; HB, health behavior; HF, health factor.

### 3.2 Survival analysis

The survival status of the population was presented in Table 2. The unweighted and weighted total deaths/participants were 416/1593 and 1,941,021/9,208,187, respectively. The all-cause mortality rate per 1,000-person year were 31.50 (95% CI 31.26–31.75). They were lower in patients with moderate [29.11 (95% CI 28.82–29.40)] and high LE8 scores [12.42 (95% CI 11.49–13.35)] than in those with low LE8 scores [47.93 (95% CI 47.37–48.49)].

**Table 2:** Survival status of participants according to total Life’s Essential 8 score.

Variables	Low score	Moderate score	High score	Total
Deaths/participants (unweighted)	118/388	278/1112	20/93	416/1593
Deaths/participants (weighted)	561,645/1,930,507	1,325,023/6,759,852	5,4,353/517,828	1,941,021/9,208,187
Mortality rate per 1,000 person-year	47.93 (47.37, 48.49)	29.11 (28.82, 29.40)	12.42 (11.49, 13.35)	31.50 (31.26, 31.75)

**Figure 1:**
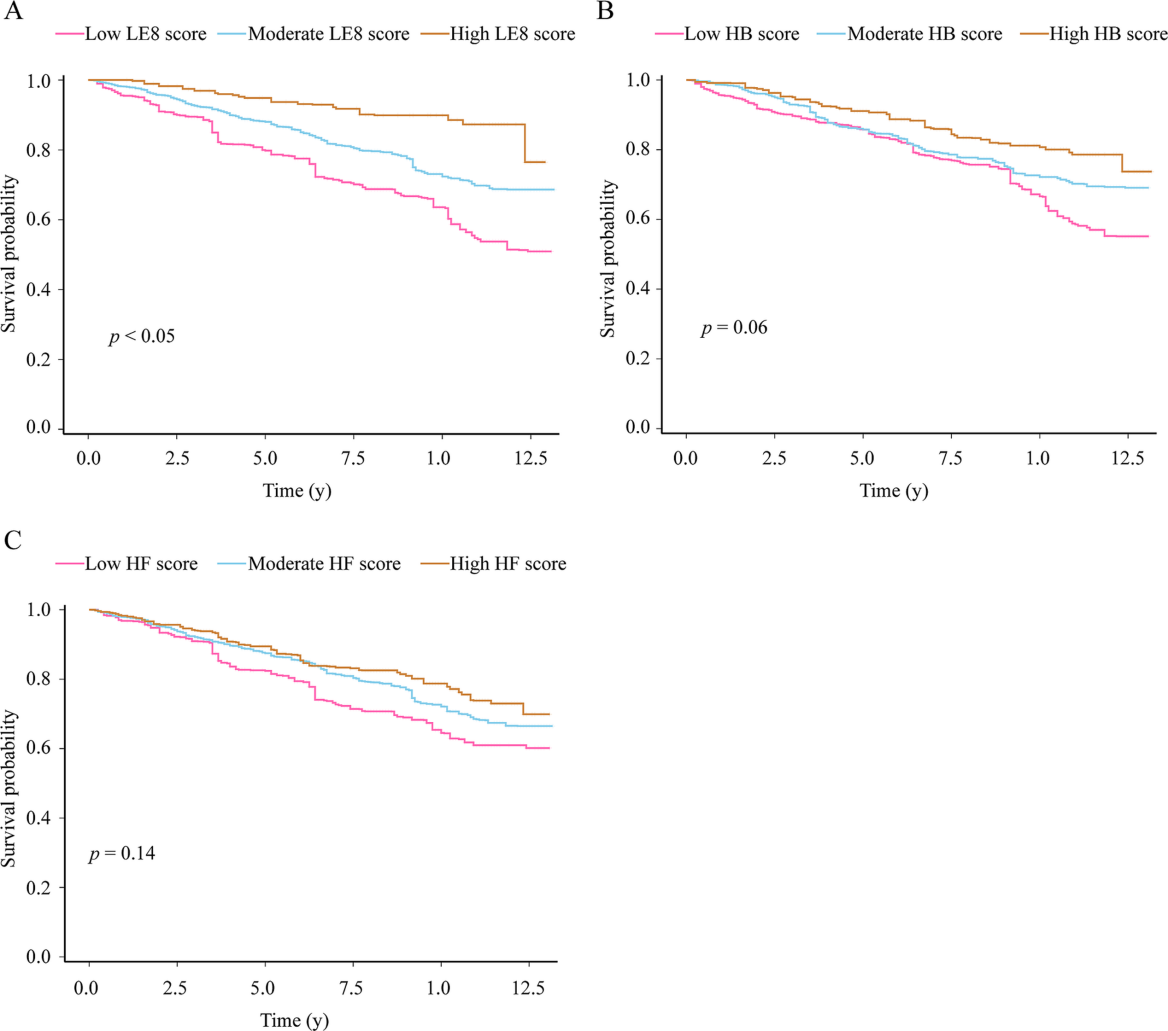
Kaplan–Meier survival curves for all-cause mortality in patients with COPD according to Life’s Essential 8 score **(A)**, health behavior score **(B)** and health factor score **(C)**. The differences were examined by log-rank test. LE8, Life’s Essential 8; HB, health behavior; HF, health factor.

The Kaplan–Meier curve of all-cause death was shown in Figure 1. The median follow-up was 5.8 years in the study. The median survival time was not achieved in the three groups. Mortality was different among patients in different LE8 score groups (log-rank *p*-values < 0.05). Patients in both the moderate and high score groups had lower mortality rates than those in the low group (both log-rank *p*-values < 0.05). The difference was not statistically significant among the three HB and HF score groups (both log-rank *p*-values > 0.05). Compared with participants with low HB scores, those with high HB scores had decreased all-cause mortality (log-rank *p*-values < 0.05). Additionally, the 10-year survival rates were 63.6, 72.4 and 89.9% for patients with COPD in the low, moderate and high LE8 score groups, respectively.

### 3.3 Association of LE8 with mortality

**Figure 2:**
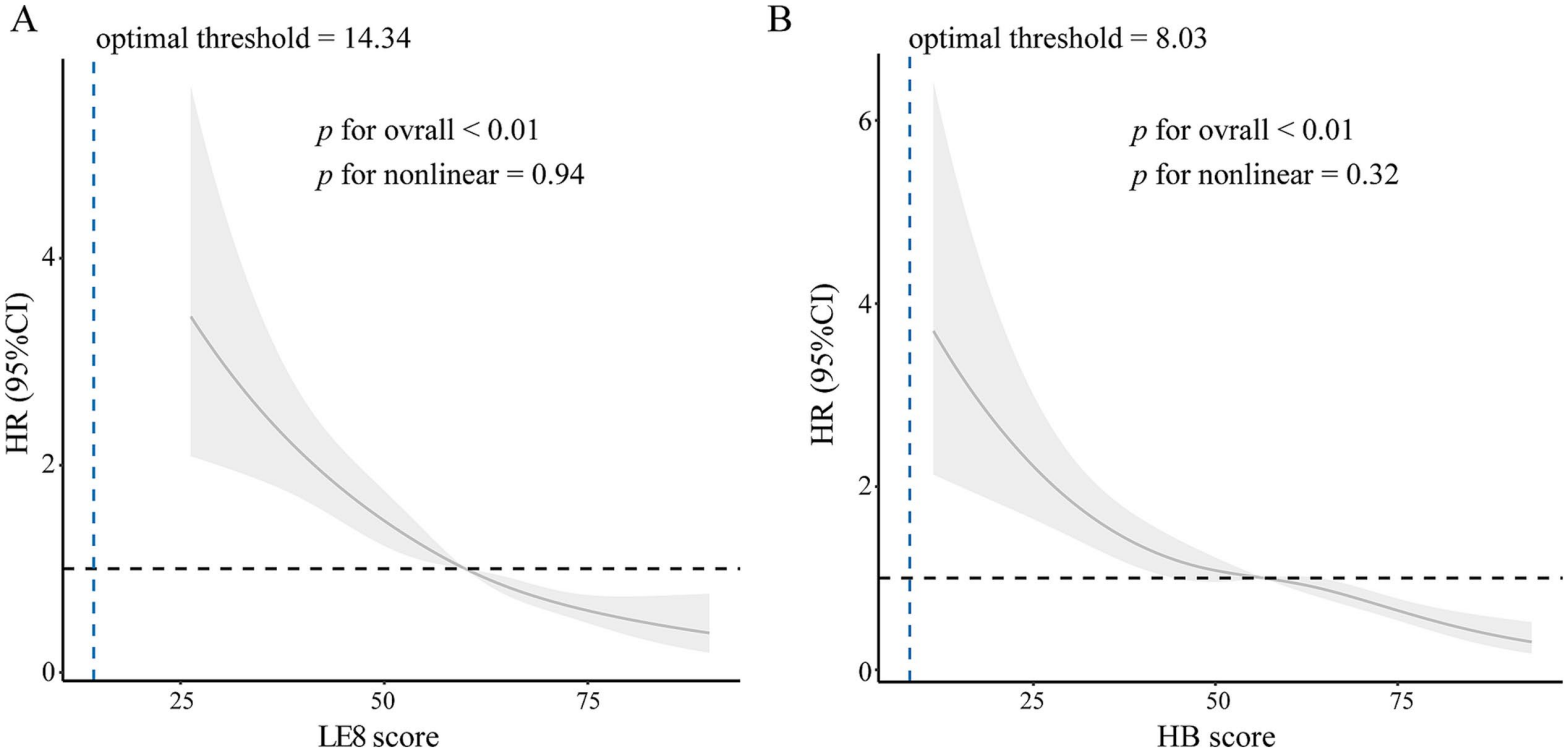
Dose–response relationships between total score **(A)** and health behavior score **(B)** and all-cause mortality. Hazard ratios (solid lines) and 95% confidence intervals (shaded areas) were adjusted for age. The blue dashed line represented the optimal threshold. LE8, life’s essential 8. HR, hazard ratio; HB, health behavior.

The hazard ratio (HR) of LE8 score with risk of all-cause mortality were shown in Table 3. The age-adjusted risk of all-cause mortality was lower in patients with high (HR 0.21, 95% CI 0.11–0.38) and moderate (HR 0.58, 95% CI 0.42–0.80) LE8 score than in those with low score. After adjustment for potential covariates, including age and education, the risk of all-cause mortality was reduced 81% (HR 0.19, 95% CI 0.09–0.40) in patients with high LE8 scores compared with those with low scores. The HR of all-cause mortality for every 10 points increase in the LE8 score was 0.77 (95% CI 0.68–0.86). There were approximately linear dose–response associations between total LE8 score and all-cause mortality risk when LE8 score was higher than 14.34 (*p* for overall < 0.01, *p* for nonlinear association > 0.05, Figure 2A). That is, the risk of all-cause mortality decreased linearly as the total LE8 score increased.

**Table 3:** Hazard ratios of Life’s essential 8, health behavior and health factor scores with the risk of all-cause mortality, NHANES 2003–2018.

Variables	Univariable model		Multivariable model 1		Multivariable model 2	
	HR (95% CI)	*p* value	HR (95% CI)	*p* value	HR (95% CI)	*p* value
LE8 score						
Low	1 (Reference)	/	1 (Reference)	/	1 (Reference)	/
Moderate	0.60 (0.43, 0.84)	0.003	0.58 (0.42, 0.80)	0.001	0.61 (0.43, 0.86)	0.005
High	0.25 (0.13, 0.46)	<0.001	0.21 (0.11, 0.38)	<0.001	0.19 (0.09, 0.40)	<0.001
Per 10 points increase	0.77 (0.69, 0.85)	<0.001	0.75 (0.67, 0.83)	<0.001	0.77 (0.68, 0.86)	<0.001
HB score						
Low	1 (Reference)	/	1 (Reference)	/	1 (Reference)	/
Moderate	0.76 (0.56, 1.04)	0.084	0.67 (0.50, 0.89)	0.007	0.68 (0.50, 0.91)	0.009
High	0.51 (0.32, 0.82)	0.005	0.41 (0.26, 0.64)	<0.001	0.53 (0.32, 0.88)	0.014
Per 10 points increase	0.87 (0.81, 0.93)	<0.001	0.81 (0.76, 0.87)	<0.001	0.83 (0.77, 0.89)	<0.001
HF score						
Low	1 (Reference)	/	1 (Reference)	/	1 (Reference)	/
Moderate	0.73 (0.51, 1.06)	0.095	0.77 (0.53, 1.12)	0.174	0.82 (0.56, 1.21)	0.325
High	0.61 (0.39, 0.94)	0.026	0.72 (0.45, 1.14)	0.158	0.74 (0.45, 1.22)	0.239
Per 10 points increase	0.86 (0.78, 0.95)	0.003	0.89 (0.79, 1.00)	0.041	0.90 (0.80, 1.02)	0.101

HR, hazard ratio; CI, confidence interval; LE8, Life’s Essential 8; HB, health behavior; HF, health factor.

Model 1 adjusted for age.

Model 2 adjusted for age and education.

Like the total LE8 score, the HB score was also associated with all-cause mortality risk (Table 3). Compared with participants with low HB scores, those with moderate and high scores had 32% (HR 0.68, 95% CI 0.50–0.91) and 47% (HR 0.53, 95% CI 0.32–0.88) lower risk of all-cause mortality, respectively, after adjustment for potential covariates. Every 10 HB score reduced all-cause mortality risk by 17% (HR 0.83, 95% CI 0.77–0.89). Similarly, a linear dose–response association was also observed between the HB score and all-cause mortality risk when HB score was higher than 8.03 (*p* for overall < 0.01, *p* for nonlinear association > 0.05, Figure 2B). However, the HF score had no significant effect on all-cause mortality risk. Furthermore, we analyzed the relationships between individual components of LE8 and mortality risk (Table 4). High scores for physical activity, smoking, sleep and blood glucose also reduced all-cause mortality risk. In contrast, diet, blood pressure, blood lipid and BMI had no effect.

**Table 4:** Hazard ratios of individual metric scores with the risk of all-cause mortality, NHANES 2003–2018.

Variables	Univariable model		Multivariable model 1		Multivariable model 2	
	HR (95% CI)	*p* value	HR (95% CI)	*p* value	HR (95% CI)	*p* value
Diet						
Low	1 (Reference)	/	1 (Reference)	/	1 (Reference)	/
Moderate	0.87 (0.65, 1.16)	0.342	0.73 (0.55, 0.96)	0.024	0.78 (0.59, 1.04)	0.089
High	0.43 (0.16, 1.16)	0.095	0.35 (0.13, 0.91)	0.031	0.40 (0.15, 1.06)	0.065
Per 10 points increase	0.94 (0.85, 1.04)	0.204	0.87 (0.79, 0.96)	0.005	0.91 (0.82, 1.00)	0.057
Physical activity						
Low	1 (Reference)	/	1 (Reference)	/	1 (Reference)	/
Moderate	0.85 (0.34, 2.13)	0.736	0.85 (0.35, 2.06)	0.719	0.86 (0.35, 2.11)	0.744
High	0.45 (0.33, 0.60)	<0.001	0.51 (0.38, 0.68)	<0.001	0.54 (0.40, 0.74)	<0.001
Per 10 points increase	0.92 (0.89, 0.95)	<0.001	0.93 (0.90, 0.96)	<0.001	0.94 (0.91, 0.97)	<0.001
Nicotine exposure						
Low	1 (Reference)	/	1 (Reference)	/	1 (Reference)	/
Moderate	1.52 (1.11, 2.07)	0.009	0.99 (0.70, 1.38)	0.941	1.06 (0.76, 1.48)	0.721
High	0.62 (0.38, 1.01)	0.056	0.45 (0.29, 0.72)	<0.001	0.51 (0.31, 0.84)	0.008
Per 10 points increase	1.01 (0.97, 1.04)	0.750	0.96 (0.93, 0.99)	0.021	0.97 (0.94, 1.01)	0.096
Sleep						
Low	1 (Reference)	/	1 (Reference)	/	1 (Reference)	/
Moderate	0.78 (0.53, 1.16)	0.215	0.62 (0.42, 0.93)	0.021	0.65 (0.44, 0.97)	0.036
High	0.79 (0.56, 1.11)	0.166	0.62 (0.45, 0.86)	0.004	0.65 (0.48, 0.92)	0.014
Per 10 points increase	0.96 (0.92, 1.01)	0.122	0.94 (0.89, 0.98)	0.006	0.95 (0.90, 0.99)	0.022
BMI score						
Low	1 (Reference)	/	1 (Reference)	/	1 (Reference)	/
Moderate	0.75 (0.53, 1.08)	0.119	0.76 (0.54, 1.06)	0.108	0.76 (0.54, 1.07)	0.114
High	0.88 (0.62, 1.25)	0.482	1.04 (0.73, 1.46)	0.839	1.05 (0.75, 1.48)	0.771
Per 10 points increase	0.97 (0.93, 1.02)	0.222	0.98 (0.94, 1.03)	0.492	0.98 (0.94, 1.03)	0.505
Blood lipids						
Low	1 (Reference)	/	1 (Reference)	/	1 (Reference)	/
Moderate	0.95 (0.62, 1.47)	0.834	0.91 (0.60, 1.39)	0.673	0.94 (0.62, 1.43)	0.782
High	1.29 (0.93, 1.78)	0.121	1.12 (0.83, 1.52)	0.452	1.16 (0.86, 1.57)	0.330
Per 10 points increase	1.03 (0.98, 1.08)	0.316	1.01 (0.96, 1.06)	0.675	1.02 (0.97, 1.07)	0.536
Blood glucose						
Low	1 (Reference)	/	1 (Reference)	/	1 (Reference)	/
Moderate	0.58 (0.40, 0.85)	0.005	0.65 (0.45, 0.94)	0.024	0.65 (0.45, 0.95)	0.027
High	0.40 (0.29, 0.56)	<0.001	0.51 (0.36, 0.72)	<0.001	0.55 (0.38, 0.79)	0.001
Per 10 points increase	0.88 (0.84, 0.92)	<0.001	0.91 (0.86, 0.95)	<0.001	0.91 (0.87, 0.97)	0.001
Blood pressure						
Low	1 (Reference)	/	1 (Reference)	/	1 (Reference)	/
Moderate	0.55 (0.37, 0.80)	0.002	0.66 (0.44, 0.98)	0.042	0.68 (0.45, 1.01)	0.059
High	0.50 (0.33, 0.76)	0.001	0.63 (0.40, 0.99)	0.044	0.67 (0.42, 1.07)	0.091
Per 10 points increase	0.93 (0.88, 0.98)	0.005	0.95 (0.90, 1.00)	0.053	0.96 (0.91, 1.01)	0.107

HR, hazard ratio; CI, confidence interval.

Model 1 adjusted for age.

Model 2 adjusted for age and education.

### 3.4 Subgroup analysis

**Figure 3:**
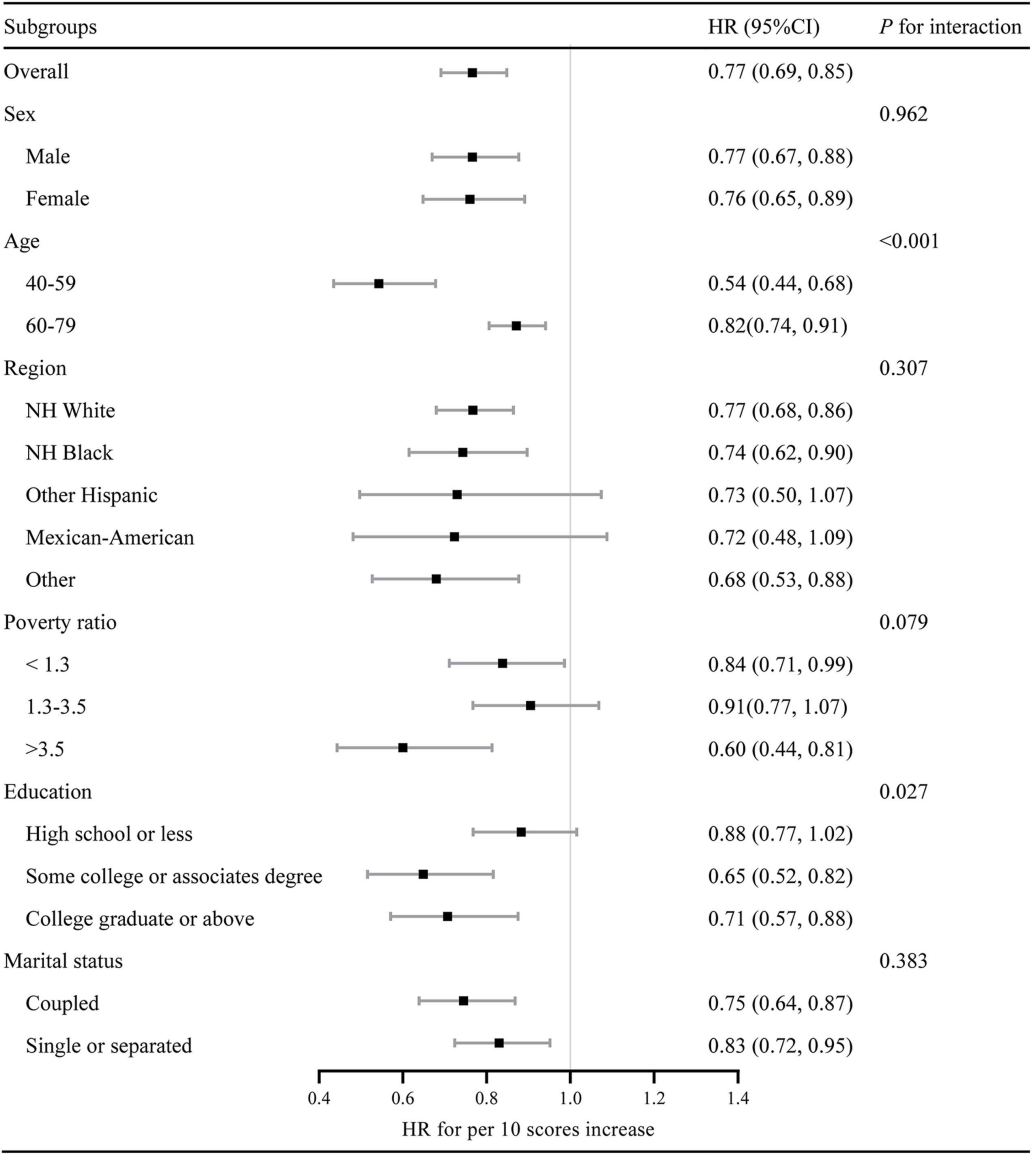
Subgroup analysis of the association between the Life’s Essential 8 scores and all-cause mortality. Hazard ratio was calculated as per 10 scores increase in the Life’s Essential 8 score. HR, hazard ratio; LE8, Life’s Essential 8.

The subgroup analyses were presented in Figure 3. The LE8 score (per 10 points) was negatively associated with all-cause mortality in all subgroups. There was an interaction between the LE8 score and age and education level. The inverse association between the LE8 score and mortality appeared to be stronger in younger patients (aged 40–60 years; HR per 10 scores increase 0.54, 95% CI 0.44–0.68), participants with some college or associate degree (HR per 10 scores increase 0.65, 95% CI 0.52–0.82), and the high-income population (poverty ratio > 3.5; HR per 10 scores increase 0.60, 95% CI 0.44–0.81).

## 4 Discussion

Based on a nationally representative cohort of the US population, we found that patients with COPD with high or moderate LE8 scores had a reduced risk of all-cause mortality. Lifestyles with high LE8 scores may benefit patients with COPD.

The healthy and favorable lifestyle of patients with COPD is a hot topic (8). However, there are currently no specific recommendation. CVD is the most common comorbidity among patients with COPD. CVD and COPD can mutually promote each other (4). Therefore, promoting CVH is crucial. LE8 is constructed to evaluate, monitor and motivate CVH. There is a reverse relationship between the LE8 score and all-cause mortality not only in patients with CVD (9), but also in patients with chronic kidney disease (10), cancer (11) and type 2 diabetes (12). In 1593 US patients with COPD, which represented 9,208,187 people, we observed a similar relationship. Furthermore, there was an approximately linear dose–response relationship between the LE8 score and all-cause mortality when LE8 score was higher than 14.34. Because of the small sample size below the threshold, we were unable to further confirm the relationship when LE8 score was lower than 14.34. Above the threshold, a 10-point increase in the LE8 score can reduce the mortality risk by 23%. Therefore, changing lifestyles to improve the LE8 score may be a potential strategy to reduce the mortality of patients with COPD. Unfortunately, only 5.6% of patients achieved high scores. It is time to take measures.

LE8 consists of HB and HF. The results indicated that patients with high HB scores had a 47% lower mortality risk than those with low score. HB contains 4 elements: diet, nicotine, sleep and physical activity. Among these factors, physical activity is an important contributor to decreased mortality risks, which was consistent with previous findings (13). Compare with those with low scores, all-cause mortality risk was 46% lower in patients with high physical activity scores. So, a patient who perform 150 min of moderate or high intensity activity per week have a 46% lower mortality risk than those who do not do any moderate or higher intensity activity. However, patients may not benefit obviously from short-time exercise. Physical activity has been a hot topic in recent years. The GOLD guidelines recommend regular physical activity for all patients (13). Here, we further scored physical activity on the basis of duration, which provided more specific advice. However, it may not be realistic for patients experiencing hypoxia and dyspnea or with severe limitations to tolerate moderate or strong intensity activity. Studies showed that patients with moderate-to-severe COPD can benefit from light-intensity activities (14) or increasing steps per day (15). Thus, for those with severe limitations, it would be valuable to modify the scoring criteria and develop a personalized exercise program. Moreover, fear of breathlessness and lack of motivation are obstacles that prevent patients from being more physically active (16). Therefore, psychological guidance, education and encouragement are crucial to encourage patients to move.

Sleep health is another important contributor. Previous studies have indicated that sleep disorders correlated with greater COPD severity (17). Patients with impaired sleep are less likely to achieve symptom improvement and have increased risk of future exacerbation (18). Our study further revealed that mortality risk was 35% lower in patients with moderate sleep scores than in those with low scores, indicating that patients can benefit obviously from a good sleep. However, sleep disorders are common among COPD patients (19). One of the main reasons is that COPD symptoms, such as cough and dyspnea, can easily affect sleep quality. Therefore, active and effective measures should be taken to improve sleep.

Smoking is a well-known major risk factor for the development of COPD. Smoking time is associated with mortality in patients with COPD (20), and smoking cessation can slow the progression of COPD (21). Here, we first demonstrated that a high nicotine score reduced mortality risk by 49% compared with a low score, and it contribute most to mortality reduction. However, moderate scores had no significant effect. Thus, patients who quit smoking for more than 1 year but for less than 5 years had the same risk of death as current smokers. However, if individuals quit smoking for more than 5 years, their mortality risk can decrease by 49% compared with that of current smokers. Therefore, persuade and help smokers to give up smoking as early as possible.

Among the four HF, blood glucose health was an important factor that was inversely associated with mortality risk. The results revealed that patients with high blood glucose scores had a 45% lower mortality risk than those with low score. Previous evidence has also indicated the negative effect of high blood glucose on lung function (7). Therefore, it is crucial to strengthen blood glucose management in patients with COPD.

There was no relationship between blood lipids or BMI and mortality risk. These findings conflict with those of a previous study in which low LDL cholesterol and underweight BMI increased mortality risks (22–24). Moreover, in a study of patients with COPD with excess weight, lifestyle intervention to reduce weight failed to improve the physical functional status and dyspnea of patients (25). Thus, high-quality evidence is necessary to further elucidate the relationship between BMI and lipid and mortality risks of patients with COPD.

Increasing evidence shows that diet and nutrition are risk factors for COPD development (6). Unexpectedly, HEI-2015 score, a dietary model suggested by the AHA, had no impact on mortality risks. Since patients with COPD are always accompanied by complicated physical conditions, such as decreased muscle mass and altered body composition, a specific dietary model should be developed for these patients. In addition, blood pressure had no effect on mortality risk. A possible explanation is the “U-shaped” relationship between blood pressure and mortality risk in patients with COPD. In a multicenter prospective trial that included 16,485 participants with COPD, both high and low blood pressure were associated with increased all-cause mortality and cardiovascular events (26). A blood pressure less than 120/80 mmHg was scored as 100 according to the AHA recommendation. Thus, adjusted scoring criteria for blood pressure may also be necessary.

There are several limitations in the present study. First, confirmation of the diagnosis of COPD was based on questionnaire, with no available spirometry results. However, this definition has been addressed and validated in several previous studies. Second, lung function, medication use, COPD severity, COPD phenotype and comorbidity, strong confounding factors for mortality risk in patients with COPD, were not adjusted, since pulmonary function tests were not performed in most patients and there is a lack of more detailed clinical information in the database. Future studies need to further analyze the effect of these potential confounders on the association of LE8 score with mortality risks and developed more personalized scoring system. Third, the relationships between diet, blood pressure, blood lipids and BMI were unexpected. While we did not further explore how to scoring these four metrics is more applicable for COPD patients. Fourth, as we discussed above, patients with COPD, especially those with poor lung function, experienced more complicated pathological and physiological changes. So, it is necessary to modify the scoring system for them. Fifth, participants of the study are mainly NH white and NH black. Whether these findings are consistent in other races need to be further investigated.

The present study highlights lifestyle modification in patients with COPD. A shift toward a comprehensive health management approach involving multidisciplinary teams could be recommended. Patients could be educated to take more active role in managing their lifestyle to improve their LE8 scores. Additionally, the score could also be integrated into risk assessment models to better predict patient prognosis and guide personalized treatment plans. Future researches should further explore the causal relationship between these lifestyle factors and mortality risk, acute exacerbation, lung function decline and quality of life in patients with COPD through prospective randomized controlled trials. A personalized scoring system should be developed to benefit more patients with different severities. It will also be necessary to investigate the underlying biological mechanisms, such as the effect of glucose metabolism on airway remodeling, to further unveil the effect of these factors on COPD.

## 5 Conclusion

Consequently, higher LE8 scores were found to be associated with a lower risk of all-cause mortality. Managing lifestyle to improve LE8 scores may be an applicable and effective approach for increasing the longevity of patients with COPD. An adjusted LE8 score may be more applicable and beneficial for patients with COPD.

## Data Availability

Publicly available datasets were analyzed in this study. This data can be found here: https://wwwn.cdc.gov/nchs/nhanes/Default.aspx.

## Footnotes

^1^ https://wwwn.cdc.gov/nchs/nhanes/tutorials/Weighting.aspx
